# ACE2 Protein Expression During Childhood, Adolescence, and Early Adulthood

**DOI:** 10.1177/10935266221075312

**Published:** 2022-02-28

**Authors:** Bernadette Schurink, Eva Roos, Wim Vos, Marjolein Breur, Paul van der Valk, Marianna Bugiani

**Affiliations:** 1 1209 Department of Pathology, Amsterdam University Medical Centers, VU University, Amsterdam, the Netherlands

**Keywords:** ACE2, children, SARS-CoV-2, immunohistochemistry

## Abstract

**Purpose and context**. Angiotensin-converting enzyme 2 is the entry receptor for SARS-CoV and SARS-CoV-2. Variations in ACE2 expression might explain age-related symptomatology of COVID-19, that is, more gastro-intestinal symptoms and less pulmonary complaints. This study qualitatively investigated ACE2 protein expression in various organs from the fetal to the young adolescent stage. **Method**. Autopsy samples from lung, heart, liver, stomach, small intestine, pancreas, kidney, adrenals, and brain (when available) were obtained from twenty subjects aged 24 weeks gestational age through 28 years. Formalin-fixed paraffin-embedded 4-um-thick tissue sections were stained against ACE2. **Key results**. We showed that the extent of ACE2 expression is age-related. With age, expression increases in lungs and decreases in intestines. In the other examined organs, ACE2 protein expression did not change with age. In brain tissue, ACE2 was expressed in astrocytes and endothelial cells. **Conclusions**. Age-related ACE2 expression differences could be one substrate of the selective clinical vulnerability of the respiratory and gastro-intestinal system to SARS-CoV-2 infection during infancy.

## Introduction

Coronavirus disease 19 (COVID-19) has been a global pandemic for almost 2 years. During this year, many of its mysteries have been unraveled. Interestingly, children seem less affected than adults and when infected, present with different symptomatology, that is, more gastro-intestinal symptoms and less pulmonary complaints.^1,2^ Reasons for this are largely unknown, but might be attributable to different expression of angiotensin-converting enzyme 2 (ACE2) between children and adults. ACE2 is a metallopeptidase also functioning as receptor for SARS-CoV and SARS-CoV-2,^3,4^ responsible for COVID-19.

Although in adults the presence of ACE2 mRNA has been established in virtually all organs,^5^ studies on its age-related expression are limited^6-11^ and mainly used RNA sequencing to assess ACE2 localization. However, mRNA and protein expression patterns do not necessarily correspond due to processes as post-translational modification. Furthermore, the use of immunostaining is capable of showing ACE2 expression in different subsets of cells that make up the organ parenchyma. Insight into the ACE2 protein expression profile during development and childhood is of great importance in understanding SARS-CoV-2 pathogenesis and age-related symptomatology of COVID-19. Therefore, the present study qualitatively investigated ACE2 protein expression in various organs from fetuses, younger and older children, adolescents, and young adults.

## Materials and Methods

The cohort consisted of twenty subjects aged 24 weeks gestational age through 28 years, who underwent autopsy for diagnostic reasons (see Table 1 for baseline characteristics). Informed consent for research purposes was obtained by the next of kin of all patients. The study was approved by the Ethical Medical Committee of the VU University Medical Center and conducted according to the Declaration of Helsinki. All cases preceded the COVID-19 pandemic; sepsis cases were excluded to avoid changes in expression of ACE2.^12^ Formalin-fixed paraffin-embedded 4-um-thick tissue sections were obtained from the lung, heart, liver, stomach, small intestine, pancreas, kidney, adrenals, and brain when available. Sections were stained against ACE2 (HPA Atlas, HPA000288, 1:500, incubated for 48 minutes) in antibody diluent (Dako, S3022) after retrieval in cell conditioning 1 solution (24 minutes, Ventana Medical Systems, 950-124) using a Ventana Benchmark Ultra machine (48 minutes, Roche) and developed with OptiView DAB IHC detection kit (Roche, 760-700). Cellular localization of ACE2 protein was assessed and immunoreactivity intensity qualitatively appraised by 2 histopathologists (PV and MB).

**Table 1.** Age and Cause of Death.

N	Age at Death	Cause of Death	Hardy Scale	Time to Autopsy (in days)	Ventilation
1	24+3 weeks GA	Intrauterine fetal demise and growth retardation, placental insufficiency	3	3, 2	No
2	30+2 weeks GA	Intrauterine fetal demise, placental infarction	3	3, 2	No
3	39+3 weeks GA	Intrauterine fetal demise of unknown cause	3	0, 8	No
4	1 day	Pulmonary hypertension with alveolar-capillary dysplasia and large-vessel misalignment	0	0, 9	Yes
5	9 days	Alveolar-capillary dysplasia without misalignment	0	0, 7	Yes
6	2 months	Intracerebral hemorrhage	0	0, 8	Yes
7	5 months	Positional asphyxia	2	0, 8	No
8	7 months	Hypoxic ischemic encephalopathy	3	0, 5	No
9	22 months	Myocarditis	0	0, 7	Yes
10	2 years	Intracerebral hemorrhage	0	1, 6	Yes
11	2 years	Myocarditis with gastroenteritis	3	3, 1	No
12	5 years	Rhabdomyolysis (*LPIN1* mutation)	0	0, 5	Yes
13	11 years	Osteosarcoma	4	0, 6	No
14	16 years	Myocarditis	3	0, 1	No
15	18 years	Myocarditis	0	0, 5	Yes
16	23 years	Metastasized testicular seminoma	0	2, 3	Yes
17	24 years	Acute myeloid leukemia	0	0, 1	Yes
18	26 years	Drug overdose	3	1, 8	No
19	26 years	Myocarditis	3	2, 5	No
20	28 years	Drug overdose	3	1, 8	No

Abbreviation: GA: gestational age.

Hardy scale: (1) Violent and fast death (terminal phase < 10min); (2) fast death of natural causes (terminal phase estimated at < 1 hr); (3) intermediate death after a terminal phase of 1 to 24; (4) slow death after a long illness, with a terminal phase longer than 1 day; (0) ventilator case, all cases on a ventilator preceding death. (GTEx Consortium. Hardy scale description. [cited 6 Dec 2021]. Available: https://www.ncbi.nlm.nih.gov/projects/gap/cgi-in/variable.cgi?study_id=phs000424.v4.p1&phv=169092#:∼:text=Deathclassificationbasedonthe,phaseestimatedat%3C10min).

## Results

**Figure 1.**
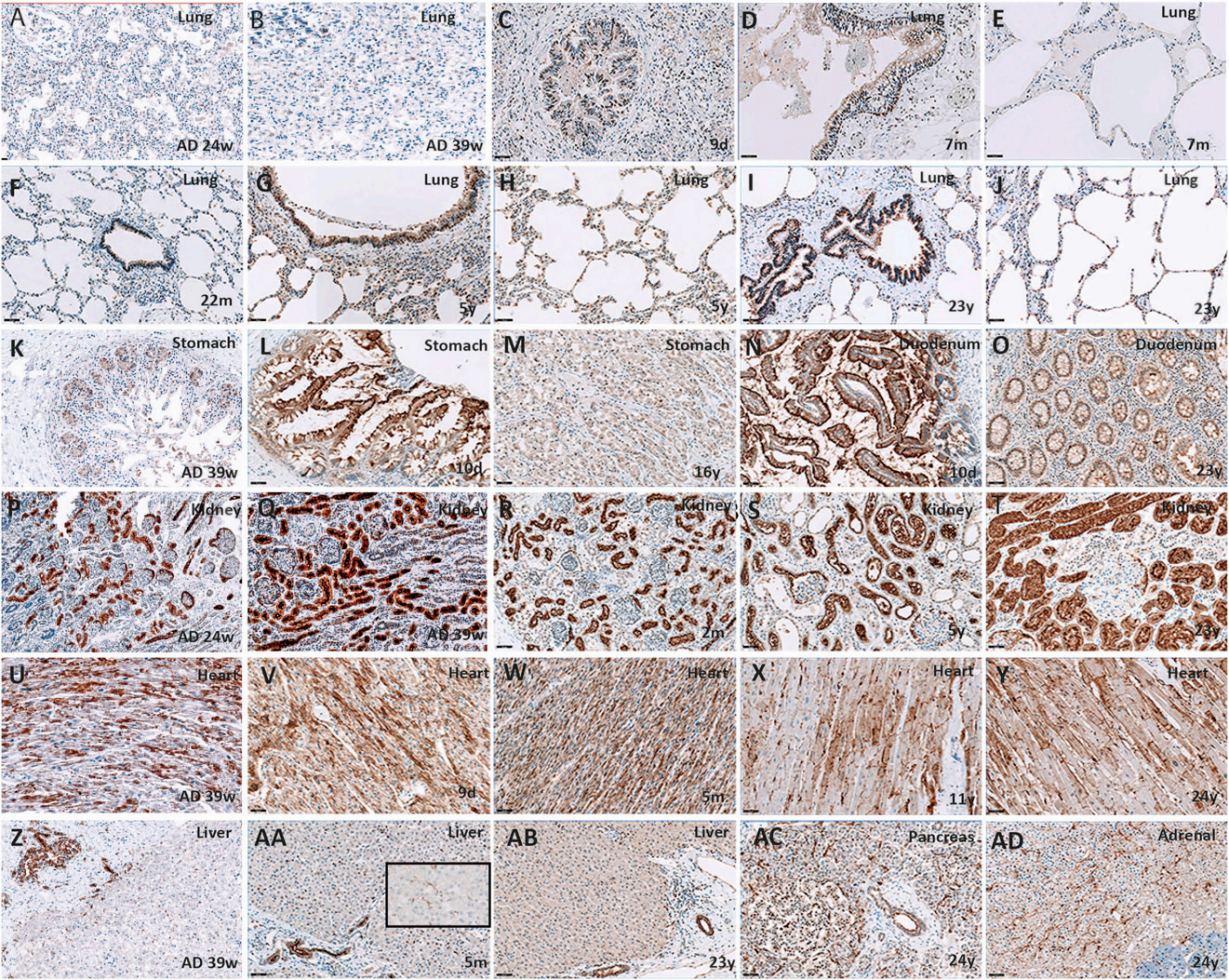
ACE2 *protein expression through ages. All magnification ×200* (A-J). In the lungs, ACE2 protein expression increases with age in both bronchiolar epithelium and alveolar pneumocytes. (K-M) In stomach mucosal epithelial cells, ACE2 protein expression is enriched at the luminal surface and decreases with age. (N and O) The same occurs in the duodenum. (P-T) In the kidney, ACE2 protein expression is found in tubuli and epithelial blade of the Bowman’s capsule, and does not change with age. (U-Y) ACE2 protein expression in the heart is found in cardiomyocytes with enrichment at the Z-line. It remains stable through ages. (Z-AB) In the liver, ACE2 protein expression in the bile duct and Kupffer cells (insert) does not change with age. The same occurs in the pancreas (AC) and adrenal gland (AD). Note the nuclear localization in many cell types. ACE2 protein may modulate reactive oxygen species formation in the nucleus, providing a protective mechanism against oxidative stress and cell damage.^7^

ACE2 showed various subcellular staining patterns (Table 2) and was expressed in various organs. The figure illustrates the results of ACE2 protein expression in different organs (Figure 1). Endothelium of blood vessels was ACE2 protein-immunoreactive in all organs at all ages. ACE2 protein expression in the lungs was detected in type I and II pneumocytes, intra-alveolar macrophages, and respiratory epithelium of large and small bronchioles. Expression increased with age to stabilize from 5 years on. ACE2 protein expression in the stomach was confined to epithelial and ganglion cells and strongly decreased with age. The same ACE2 protein spatial and temporal expression pattern was found in the small intestine. In the other examined organs, ACE2 protein expression did not change with age (cardiomyocytes, liver Kupffer cells and bile duct epithelium, kidney tubular and Bowman capsule epithelium, adrenal cortex, pancreatic acini and ductular epithelial cells, and endocrine cells with capillary endothelium; not shown, but available from authors upon request).

**Table 2.** ACE 2 Location and Subcellular Staining Patterns.

Organ	Cellular Location	Stain Location		
		Nuclear	Cytoplasmatic	Membranous
Lung	Pneumocytes	+	+	-
	Macrophages	-	+	-
	Respiratory epithelium	+	+	+
Heart	Cardiomyocytes	-	+	+ (also Z-line)
Stomach	Follicular and glandular epithelium	-	+	+
	Ganglion cells	+	-	-
Small intestine	Epithelium	-	+	+
	Lymphoid tissue	+	-	-
	Ganglion cells	+	-	-
Liver	Kupffer cells	+	+	-
	Bile ducts	-	+	+
	Hepatocytes	Weak, negative	Weak, negative	-
Pancreas	Acini (sporadically)	-	-	+
	Ducts	-	-	+
Kidneys	Cortical tubuli^a^	-	+	+
	Bowman’s capsule	+ (endothelium)	+ (epithelium, endothelium)	-
Adrenal glands	Zona glomerulosa	-	+	-
	Zona fasciculate	+	+	-
	Zona reticularis	-	+	-
Brain	Astrocytes	-	-	+

Endothelium of blood vessels was ACE2 protein-immunoreactive in all organs.

^a^Difference in expression between distal (overt expression of luminal surface and cytoplasm basal and luminal surface) and proximal (much less, cytoplasmatic and nuclear) tubuli.

In brain tissue, ACE2 was expressed in astrocytes and endothelial cells; no expression changes were detected amongst ages.

## Discussion

Our study confirms that ACE2 is widely expressed during development and across all ages, paralleling findings of mRNA studies in adults.^5^ Interestingly, we found previously unreported age-related differences in ACE2 expression in the lungs and gastro-intestinal tract, mainly present in cells in contact with the external environment (bronchiolar epithelial cells, enterocytes). ACE2 expression in the lungs increased during the first years of life, whereas ACE2 expression in the stomach and small intestine decreased with age. These age-related expression differences could be a substrate of the selective clinical vulnerability of the respiratory and gastro-intestinal system to SARS-CoV-2 infection during infancy.^1,2^ In other organs, expression of ACE2 protein did not change with age.

The majority of studies that evaluate ACE2 expression across ages were limited to the lungs. An age-dependent increase in ACE2 expression in the human lung was further found in this study, with absent staining during later gestational ages and an increase during the first years of life. This has also been reported by others^6-8,11^ and might partly explain the predominance of respiratory symptoms in older subjects infected with COVID-19. Although results are conflicting, it is important to take note of ventilation as a possible confounding factor^11,13^ in pulmonary ACE2 expression.

ACE2 expression in other organs in early life, childhood, and adolescence has not been studied before. Expression during fetal life has been investigated by few, and results are conflicting.^7,9^ This might be explained by the differences in techniques used (immunohistochemistry vs single-cell RNA sequencing) and the aforementioned difference between RNA and mRNA/protein expression. Therefore, although our sample size was limited, we are the first to present a comprehensive view on ACE2 expression during the different stages of life and illustrate that not only does ACE2 expression in the lung increase with age, ACE2 expression in the gut decreases with age. Our results could be one substrate of the selective clinical vulnerability of the respiratory and gastro-intestinal system to SARS-CoV-2 infection during infancy in comparison with adulthood.
